# Risk factors for peripherally inserted central catheter-related venous thrombosis in adult patients with cancer

**DOI:** 10.1186/s12959-023-00574-4

**Published:** 2024-01-04

**Authors:** Pinghu Wang, Lianxiang He, Qiong Yuan, Juan Lu, Qingqiong Ji, An Peng, Wanli Liu

**Affiliations:** 1 Breast Surgery Department, Hunan Provincial Maternal and Child Healthcare Hospital, Changsha, Hunan People’s Republic of China; 2 Teaching and Research Section of Clinical Nursing, Xiangya Hospital, Central South University, Changsha, Hunan People’s Republic of China; 3 Xiangya Changde Hospital, Changde, Hunan People’s Republic of China; 4 Institute for Rational and Safe Medication Practices, National Clinical Research Center for Geriatric Disorders, Xiangya Hospital, Central South University, Changsha, Hunan People’s Republic of China

**Keywords:** Peripherally inserted central catheter, Venous thrombosis, Adult patients, Cancer, Risk

## Abstract

**Purpose:**

The purpose of this study was to understand and analyze the risk factors of peripherally inserted central catheter (PICC)-related venous thrombosis in adult patients with cancer.

**Methods:**

This observational cohort study included adult patients with cancer who underwent color Doppler ultrasound at the Xiangya Hospital of Central South University, Hunan Provincial Maternal and Child Healthcare Hospital, and Xiangya Changde Hospital, Hunan Province, from January 1, 2017 to December 31, 2021. Univariate and multivariate logistic regression analyses were performed to determine the risk factors of PICC-related venous thrombosis.

**Results:**

After risk adjustment, multivariate logistic regression analysis revealed statistically significant associations between PICC-related venous thrombosis and age > 65 years old (OR: 1.791, CI: 1.343–2.389), male sex (OR: 1.398, CI: 1.057–1.849), white blood cell count > 9.5 × 10^9^ /L (OR: 1.422, CI: 1.041–1.942), APTT < 25 s (OR: 2.006, CI: 1.431–2.811), gastrointestinal tumor (OR: 2.191, CI: 1.406–3.414), infection (OR:7.619, CI: 5.783–10.037), the use of cisplatin (OR: 2.374, CI: 1.714–3.214), vincristine (OR: 2.329, CI: 1.447–3.749), the use of polyurethane (OR: 2.449, CI: 1.863–3.219) and open-ended catheters (OR:1.660, CI: 1.131–2.439), keeping time of the catheter (days) (OR: 1.003, CI: 1.001–1.005) were associated with PICC-related venous thrombosis.

**Conclusion:**

We identified that the presence of age > 65 years old, male sex, white blood cell count > 9.5 × 10^9^ /L, APTT < 25 s, gastrointestinal tumor, infection, the use of cisplatin and vincristine, the use of polyurethane, open-ended catheters and keeping time of the catheter (days), were associated with PICC-related venous thrombosis.

## Introduction

Peripherally inserted central catheters (PICCs) are widely used globally, especially in patients with cancer who commonly need PICCs to prevent peripheral vein damage during chemotherapy, bone marrow transplantation, and transfusion of blood products and their components [1]. However, tumors can activate the coagulation system, causing it to exhibit various degrees of hypercoagulable states, and PICC insertion can lead to injury to the vessel wall.

The incidence of asymptomatic PICC-related venous thrombosis in patients with cancer diagnosed by routine screening varies from 2 to 66%, while the incidence of symptomatic PICC-related venous thrombosis ranges from 2.7 to 13.8% [1–3]. A previous study showed that patients with venous thromboembolism (VTE) have a poorer prognosis than that of patients without it [4]. Further, VTE can also result in accidental decannulation and pulmonary embolism [5, 6].

The development of venous thrombosis is a multifactorial process, described in Virchow’s classic triad, including endothelial injury, venous blood flow arrest, and/or potential hypercoagulable blood. In patients with malignant tumors, the systemic mechanisms involved in the pathogenesis of cancer-related thrombosis and local factors related to vascular injury during catheter placement may be beneficial for the development of PICC-related venous thrombosis [7].

However, there is limited data on risk factors for catheter-related thrombosis [8]. In addition, although various risk assessment models have been developed to identify high-risk patients with VTE [9–11], their positive predictive value is still limited [12–13] and not suitable for PICC-related venous thrombosis. Therefore, we conducted a multicenter observational cohort study of consecutive hospitalized adult patients with PICC catheterization to determine the risk factors for PICC-related thrombosis.

## Methods

### Patients and study design

**Fig. 1:**
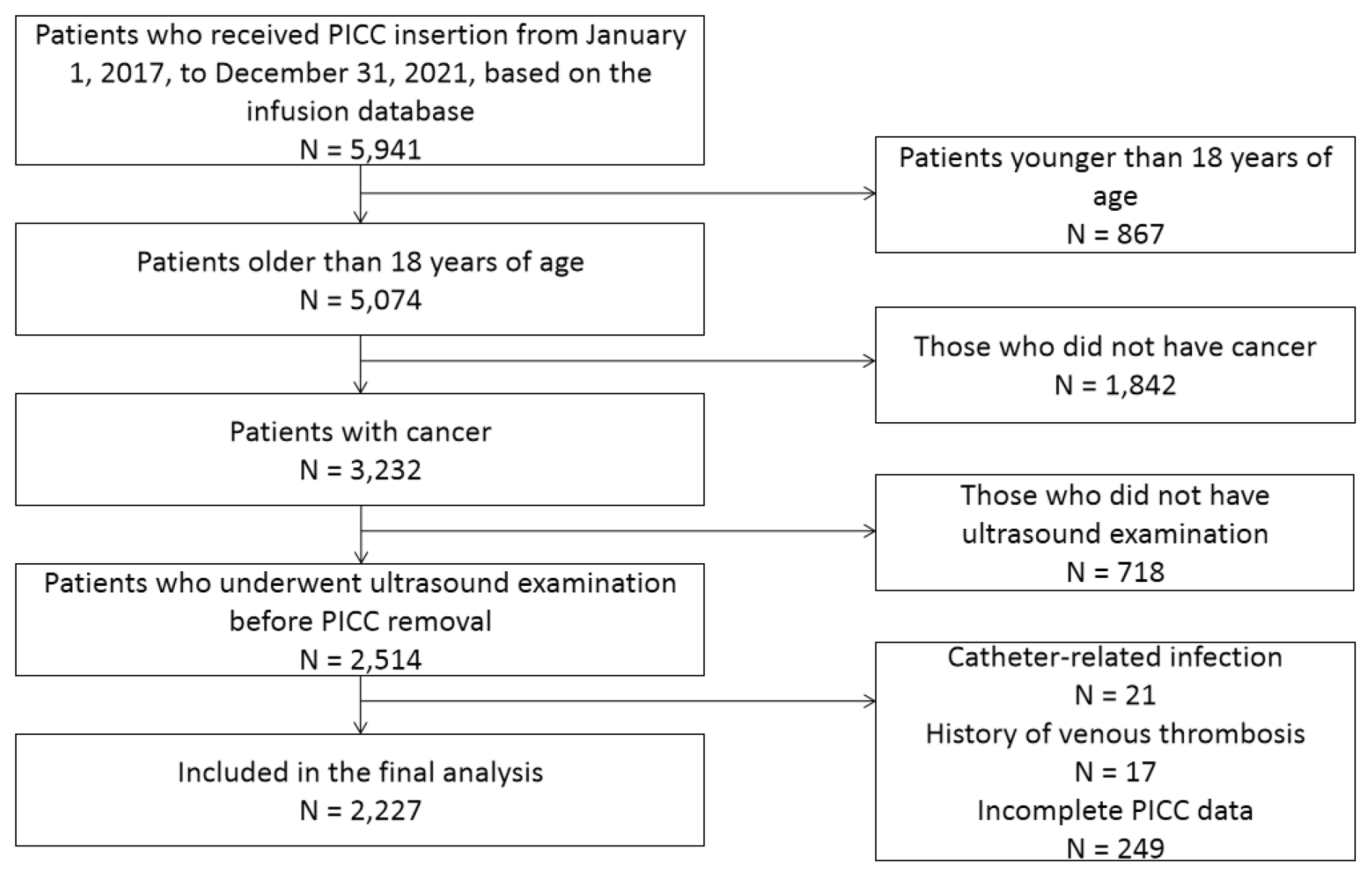
The screening process

Adult patients with cancer who received PICC insertion from January 1, 2017 to December 31, 2021 at the Xiangya Hospital of Central South University, Hunan Provincial Maternal and Child Healthcare Hospital, and Xiangya Changde Hospital, Hunan Province, China, were screened. The inclusion criteria were as follows: (l) patients who used PICC during their stay in the hospital; (2) patients diagnosed with cancer and recommended to undergo chemotherapy or long-term infusion therapy; (3) patients older than 18 years of age; and (4) patients who underwent ultrasound examination before PICC removal. The exclusion criteria were as follows: (1) confirmed or suspected catheter-related infection; (2) history of venous thrombosis; and (3) other catheters on the puncture side. A total of 5,941 PICCs were inserted in 5,917 patients at 3 tertiary hospitals in Hunan province. The final sample size included in this study was 2,227. The screening process is shown in Fig. 1.

### The PICC management policy

The PICC management primary protocol involves conducting catheter maintenance every 7 days. The PICC maintenance includes skin disinfection, flushing the catheter, replacing the dressing, and sterile connector, ensuring strict adherence to aseptic principles. The catheter can be retained for a maximum period of one year, with regular maintenance and monitoring.

### Diagnosis of PICC-related venous thrombosis

A routine examination of PICC-related thrombosis was performed using a color Doppler ultrasound (CDU) before PICC removal. CDU was used to detect the existence of thrombosis immediately if suspected symptoms of PICC-related venous thrombosis occurred, including redness, swelling, pain, or moving disorder when inserting the catheter.

CDU was performed by an experienced CDU technician using a Philips Epiq 5 (Eindhoven, Netherlands) and high-resolution linear array transducer (9 − 13 MHz). The criteria for the diagnosis of venous thrombosis were as follows: the lumen could not be compressed despite firm compression with the transducer probe, defective blood flow signal in the lumen, solid return in the lumen sound, disappearance or weakening of the spent response, phase change in the loss of blood spectrum, and disappearance or weakening of the blood flow in the distal limb by squeezing.

### Data collection

All patients were identified from our infusion database. General information on patient-related potential risk factors obtained before catheter insertion included age, sex, body mass index (BMI), white blood cell count, platelet count, hemoglobin concentration, D-dimer concentration, and activated partial thromboplastin time (APTT). We also identified whether the cases involved multiple myeloma, lung cancer, gastrointestinal tumors (such as gastric and colorectal cancer), hematologic neoplasms, metastases. We assessed the administration of chemotherapy agents, including cisplatin, carboplatin, vincristine, epirubicin, paclitaxel, and docetaxel, as well as the presence of comorbidities such as diabetes, hyperlipidemia, infection. Additionally, we assessed smoking and factors related to potential PICC-associated risks. This includes central venous catheter history, using polyurethane PICC, 5-Fr, double-lumen, open-ended and right-side, non-basilic vein catheters, catheter displacement and keeping time of the catheter (days).

### Statistical analysis

Descriptive statistics were used to evaluate the study population and the incidence of PICC-related thrombosis. All data were analyzed using SPSS for Windows (version 22.0; SPSS Inc., Chicago, IL, USA). The measurement data are expressed as mean ± standard deviation. The statistical description of enumeration data is expressed in frequency and percentage. Differences in PICC-related thrombosis rates in different groups were evaluated using univariate logistic regression. Variables that were statistically significant with a two-tailed *P* < 0.05 were retained in the final multivariable model. Multivariate logistic regression analysis was used to estimate the unadjusted odds ratios (ORs) and 95% confidence intervals (CIs) for the association between all potential risk factors and PICC-related thrombosis.

## Results

### General population data

A total of 2,227 adult patients with cancer were enrolled in this study (Table I). Among the 2,227 cases, 482 were diagnosed with PICC-related venous thrombosis by CDU in 3 hospitals between January 1, 2017 and December 31, 2021. Thrombosis-related symptoms were found in 34 (7.05%) patients, and 448 (92.95%) asymptomatic patients were detected by CDU before PICC extubation. Among them, 1,011 (45.4%) were men and 1,216 (54.6%) were women, with an age range of 18–92 years (mean [54.60 ± 12.00] years), and 432 (19.4%) were older than 65 years. Before PICC placement, all patients underwent ultrasound screening. During the indwelling period, 36 cases developed suspicious symptoms such as swelling and pain in the limb on the side of the catheter, prompting immediate ultrasound examination. Upon confirmation of PICC-related venous thrombosis, anticoagulant therapy was initiated, PICC continue to be used until treatment is completed or the catheter expires. All patients with catheters underwent an ultrasound examination again before the catheter was to be removed at the end of treatment or when the patient decided to discontinue treatment. The retention time for the PICC ranged from 7 to 364 days, with a median time of 134 days. The average indwelling time for the group with PICC-related venous thrombosis was 118.56 ± 68.258 days, whereas for the group without catheter-related venous thrombosis, the average duration was 130.60 ± 57.736 days. The demographic and clinical characteristics of patients are shown in Table 1.

**Table 1:** Demographic and clinical characteristics

Factors	PICC-related venous thrombosis	No PICC-related venous thrombosis
	n = 482	n = 1,745
Age > 65 years old	133 (27.59%)	299 (17.13%)
Male	264 (54.77%)	747 (42.81%)
BMI ≥ 25	98 (20.33%)	397 (22.75%)
White blood cell count > 9.5 × 109 /L	96 (19.92%)	221 (12.66%)
Platelet count > 350 × 109 /L	37 (7.68%)	130 (7.45%)
Hemoglobin concentration < 130 g/L	340 (70.54%)	1115 (63.90%)
D-dimer concentration > 0.5 mg/L	105 (21.78%)	278 (15.93%)
APTT < 25 s	95 (19.71%)	141 (8.08%)
Multiple Myeloma	8 (1.66%)	28 (1.60%)
Lung Cancer	130 (26.97%)	266 (15.24%)
Gastrointestinal Tumor	49 (10.16%)	86 (4.93%)
Hematologic neoplasms	80 (16.60%)	277 (15.87%)
Metastasis	151 (31.33%)	479 (27.45%)
Diabetes	49 (10.17%)	130 (7.45%)
Hyperlipidemia	19 (3.94%)	69 (3.95%)
Any infection	220 (45.64%)	133 (7.62%)
Smoking	98 (20.33%)	174 (9.97%)
Cisplatin	122 (25.31%)	183 (10.49%)
Carboplatin	120 (24.90%)	307 (17.59%)
Vincristine	41 (8.51%)	67 (3.84%)
Epirubicin	109 (22.61%)	359 (20.57%)
Paclitaxel	154 (31.95%)	388 (22.23%)
Docetaxel	56 (11.62%)	198 (11.35%)
History of central venous catheter	58 (12.03%)	177 (10.14%)
5 Fr catheter	51 (10.58%)	72 (4.13%)
Polyurethane PICC	319 (66.18%)	702 (40.23%)
Open-ended catheter	66 (13.69%)	175 (10.03%)
Right-side catheter	189 (39.21%)	685 (39.26%)
Non-basilic vein catheter	42 (8.71%)	147 (8.42%)
Catheter displacement	17 (3.53%)	36 (2.06%)
Keeping time of the catheter (days)	118.56 ± 68.258	130.60 ± 57.736

Abbreviations: PICC, peripherally inserted central catheter; BMI, body mass index; APTT, activated partial thromboplastin time

### Univariate analysis of risk factors of PICC-related venous Thrombosis

In the univariate analysis, statistically significant associations were observed between PICC-related venous thrombosis and age > 65 years old, male sex, white blood cell count > 9.5 × 10^9^/L, hemoglobin concentration < 130 g/L, D-dimer concentration > 0.5 mg/L, APTT < 25 s, lung cancer, gastrointestinal tumor, the presence of any infection, smoking, and the use of the chemotherapy agents cisplatin, carboplatin, vincristine, paclitaxel, and the use of 5-Fr, polyurethane PICC, and open-ended catheters, keeping time of the catheter (Table 2).

**Table 2:** Univariate logistic regression analysis of risk factors associated with PICC-related venous thrombosis

Factors	Univariate		
	OR	95% CI	*P*
Age > 65 years old	1.843	1.456–2.332	< 0.001
Male	1.618	1.321–1.982	< 0.001
BMI ≥ 25	0.867	0.676–1.111	0.259
White blood cell count > 9.5 × 109 /L	1.715	1.317–2.234	< 0.001
Platelet count > 350 × 109 /L	1.033	0.706–1.510	0.867
Hemoglobin concentration < 130 g/L	1.353	1.087–1.684	0.007
D-dimer concentration > 0.5 mg/L	1.470	1.143–1.890	0.003
APTT < 25 s	2.793	2.105–3.705	< 0.001
Multiple Myeloma	1.035	0.459–2.286	0.932
Lung Cancer	2.053	1.616–2.610	< 0.001
Gastrointestinal Tumor	2.183	1.513–3.149	< 0.001
Hematologic neoplasms	1.055	0.803–1.385	0.702
Metastasis	1.206	0.968–1.501	0.095
Diabetes	1.406	0.995–1.986	0.053
Hyperlipidemia	0.997	0.594–1.673	0.990
Any infection	10.177	7.912–13.091	< 0.001
Smoking	2.304	1.756–3.023	< 0.001
Cisplatin	2.893	2.239–3.737	< 0.001
Carboplatin	1.553	1.221–1.975	< 0.001
Vincristine	2.328	1.557–3.482	< 0.001
Epirubicin	1.140	0.894–1.454	0.290
Paclitaxel	1.642	1.315–2.051	< 0.001
Docetax	1.027	0.749–1.408	0.868
History of central venous catheter	1.212	0.884–1.661	0.232
5 Fr catheter	2.750	1.892–3.997	< 0.001
Polyurethane PICC	2.908	2.353–3.593	< 0.001
Open-ended catheter	1.423	1.051–1.927	0.022
Right-side catheter	0.998	0.812–1.227	0.986
Non-basilic vein catheter	1.038	0.725–1.486	0.840
Catheter displacement	1.736	0.966–3.118	0.065
Keeping time of the catheter (days)	0.996	0.995–0.998	< 0.001

Abbreviations: PICC, peripherally inserted central catheter; OR, odds ratio; CI, confidence interval BMI, body mass index; APTT, activated partial thromboplastin time

### Multivariate analysis of risk factors of PICC-related venous thrombosis

After risk adjustment, multivariate logistic regression analysis showed that age > 65 years old (OR: 1.791, CI: 1.343–2.389), male sex (OR: 1.398, CI: 1.057–1.849), white blood cell count > 9.5 × 109 /L(OR: 1.422, CI: 1.041–1.942), APTT < 25 s (OR: 2.006, CI: 1.431–2.811), gastrointestinal tumor(OR: 2.191, CI: 1.406–3.414), the presence of any infection (OR:7.619, CI: 5.783–10.037), the use of cisplatin (OR: 2.374, CI: 1.714–3.214), vincristine (OR: 2.329, CI: 1.447–3.749), the use of polyurethane (OR: 2.449, CI: 1.863–3.219) and open-ended catheters (OR:1.660, CI: 1.131–2.439), keeping time of the catheter (days) (OR: 1.003, CI: 1.001–1.005)were associated with PICC-related venous thrombosis (Table 3).

**Table 3:** Multivariate logistic regression analysis of risk factors associated with PICC-related venous thrombosis

Variable	Multivariate			Multicollinearity	
	OR	95% CI	*P*	Tolerance	VIF
Age > 65 years old	1.791	1.343–2.389	0.000	0.931	1.074
Male	1.398	1.057–1.849	0.019	0.732	1.367
White blood cell count > 9.5 × 109 /L	1.422	1.041–1.942	0.027	0.964	1.037
Hemoglobin concentration < 130 g/L	1.231	0.950–1.595	0.117	0.930	1.075
D-dimer concentration > 0.5 mg/L	1.198	0.882–1.628	0.247	0.955	1.047
APTT < 25 s	2.006	1.431–2.811	0.000	0.911	1.098
Lung Cancer	1.103	0.698–1.742	0.674	0.364	2.749
Gastrointestinal Tumor	2.191	1.406–3.414	0.001	0.922	1.084
Any infection	7.619	5.783–10.037	0.000	0.878	1.138
Smoking	1.045	0.715–1.528	0.818	0.675	1.481
Cisplatin	2.347	1.714–3.214	0.000	0.854	1.170
Carboplatin	1.192	0.779–1.824	0.419	0.431	2.322
Vincristine	2.329	1.447–3.749	0.000	0.941	1.063
Paclitaxel	1.223	0.915–1.635	0.175	0.759	1.318
5 Fr catheter	1.125	0.687–1.802	0.665	0.832	1.202
Polyurethane PICC	2.449	1.863–3.219	0.000	0.693	1.442
Open-ended catheter	1.660	1.131–2.439	0.010	0.842	1.187
Keeping time of the catheter (days)	1.003	1.001–1.005	0.005	0.986	1.014
	**Risk factors for peripherally inserted central catheter-related venous thrombosis in adult patients with cancer**	**Risk factors for peripherally inserted central catheter-related venous thrombosis in adult patients with cancer**	**Risk factors for peripherally inserted central catheter-related venous thrombosis in adult patients with cancer**	**Risk factors for peripherally inserted central catheter-related venous thrombosis in adult patients with cancer**	**Risk factors for peripherally inserted central catheter-related venous thrombosis in adult patients with cancer**

Abbreviations: PICC, peripherally inserted central catheter; OR, odds ratio; CI, confidence interval; APTT, activated partial thromboplastin time

In our study, tolerance values range from 0.364 to 0.986, all above the commonly referenced threshold of 0.1, indicating that none of the variables exhibit multicollinearity at a concerning level. It is generally considered that a Tolerance value below 0.1 may indicate a serious multicollinearity issue. The VIF values range from 1.014 to 2.749, which do not exceed the commonly used critical value of 5. This suggests that, while some multicollinearity may be present, it does not reach a level that severely affects the stability of the model. In summary, the data from our research indicate that multicollinearity exists but is not severe, potentially having limited impact on the model, which also suggests that the model is robust.

## Discussion

The presence of a catheter is the most common cause of secondary venous thrombosis in patients with cancer, which is often asymptomatic [14]. In this study, 92.95% of PICC-related venous thrombosis patients were asymptomatic. Using PICCs can cause venous thrombosis via vessel wall injury, hypercoagulability, and alterations in normal blood flow. Patients with cancer show a hypercoagulable state that can promote thrombosis [15, 16].

In terms of patient factors, we found that age > 65 years is one of the risk factors for PICC-related venous thrombosis, consistent with previous research reports [17]. Most coagulation factors (fibrinogen, factors V, VII, VIII, IX, XI, and XIII and vascular hemophilia factor) increase with age, while fibrinolytic activity decreases with age, leading to an increase in procoagulant status [18, 19]. With age, vascular endothelial dysfunction occurs, and vascular endothelial cells produce high levels of coagulants such as vascular hemophilia factor and plasminogen activator inhibitor 1. Therefore, this change leads to a decrease in nitric oxide, which plays an important role in controlling vascular function and structure, reducing the protection of vascular walls against thrombosis [20, 21].

Surprisingly, we found that male sex is an independent risk factor. We can assume that this difference is related to the type of tumor and the associated chemotherapy [22]. The most common tumor among young men is germ cell tumor, and cisplatin-based chemotherapy has a high risk of thromboembolic events [23].

Several studies support the association between elevated white blood cell counts and an increased risk of thrombotic events. For example, a study found that the risk of thrombosis significantly increased in patients with a WBC count greater than 7 × 10^^9^/L, and it became statistically significant when the WBC count exceeded 11 × 10^^9^/L [24].Additionally, a study revealed that patients suffering from deep vein thrombosis had significantly increased WBC counts and plasma C-reactive protein levels, although no correlation was found between WBC count and the thrombotic burden or the duration of symptoms [25]. Meanwhile, our study also found that APTT instead of D-dimer is an important factor in the risk of PICC-related venous thrombosis in patients with cancer. APTT is a coagulation function test indicator that reflects the comprehensive activity of coagulation factors in the endogenous coagulation pathway, especially in the first stage. Tumor-infiltrating macrophages are key mediators of anti-tumor responses, producing many cytokines that increase the synthesis and release of acute phase proteins such as fibrinogen and FVIII, tilting the hemostatic balance towards the direction beneficial for hypercoagulable blood [26]. FVIII and fibrinogen can be evaluated through the accumulation of APTT, which is a cheap and simple common laboratory test. Studies have speculated that an increase in levels of one or more related coagulation factors will shorten APTT, and a short APTT ratio can be used as a substitute indicator for the increase in levels of these factors in the “intrinsic” coagulation pathway, thereby predicting the risk of catheter-related venous thrombosis in patients with cancer [27] instead of D-dimer [28]. Gastrointestinal tumors (such as gastric and colorectal cancer) can express proteins that change the host’s systems, including platelets and leukocytes, or release procoagulant proteins into the circulation that activate the coagulation cascade or platelets, like tissue factor and podoplanin, contributing to the risk of VTE [29].

The most important finding was that compared with no infection, the presence of any infection led to the highest risk odds (OR:7.619, CI: 5.783–10.037) for PICC-related venous thrombosis. Infections have typically been correlated with VTE [30, 31] and are also a VTE risk factor for hospitalized patients with cancer [32], possibly due to a procoagulant state induced by infections [33, 34]. In a meta-analysis study, PubMed, Embase, and Cochrane Library databases were searched until July 2021, and the pooled results showed that cancer (OR: 1.74, 95% CI: 1.17 − 2.57; *P* = 0.006) and infections (OR: 2.13, 95% CI: 1.33 − 3.42; *P* = 0.002) significantly increased the occurrence of catheter-related venous thrombosis [35]. Infections can promote thrombosis by injuring the endothelium, activating the procoagulant pathway induced by tissue factors, and inhibiting the endogenous anticoagulation pathway and fibrinolysis [36, 37]. The activation of neutrophils and formation of neutrophil extracellular traps are related to venous thrombosis [38, 39]. These traps promote the initiation of platelet adhesion, activation, and aggregation, which is mediated by P-selectin [40]. As for which types of infections are more closely related to PICC-associated venous thrombosis, we will further identify and confirm in future research.

Vinblastine and Cisplatin may be more likely to cause venous thrombosis compared to other chemotherapy drugs, though the primary research and data focus on Cisplatin. Studies indicate that patients receiving a Cisplatin-based chemotherapy regimen have a higher risk of thromboembolic events. Patients treated with cisplatin-based chemotherapy regimens had a VTE incidence of 1.92% (95% CI, 1.07–2.76), compared to 0.79% (95% CI, 0.45–1.13) in those treated with non-cisplatin-based regimens. The risk of VTE was significantly increased in patients receiving cisplatin-based chemotherapy (RR, 1.67; 95% CI, 1.25–2.23; *p* = 0.01) [41]. Cisplatin-induced thrombosis mechanisms remain unclear, though some studies have proposed potential mechanisms. Cisplatin-induced endothelial damage, platelet activation, and the upregulation of prothrombotic factors are all associated with thrombogenesis [42, 43]. Cisplatin-induced renal dysfunction may also play a role; patients with renal insufficiency have a higher risk of VTE [44] Specific information regarding Vinblastine is relatively scarce, but it can be inferred that different chemotherapy drugs might affect the clotting system through various mechanisms, thereby affecting the risk of venous thrombosis. They might cause platelet aggregation, damage to the endothelial lining of vessels, or interact with other risk factors (such as surgery, radiotherapy, or personal and family history), thereby increasing the risk of venous thrombosis.

In terms of catheter factors, our study found that the polyurethane material of the catheter is more likely to cause PICC-related venous thrombosis than the silicone material, which is consistent with the literature report that in 2,270 patients, the incidence of venous thrombosis associated with silicone catheters was 0.74%, while the incidence of venous thrombosis associated with polyurethane catheters was 3.17% (*P* < 0.001) [45]. Polyurethane material has a higher stiffness, mechanical stimulation of the vascular wall, increased risk of thrombophlebitis [46], and loss of function secondary to extravasal kinking [47, 48]. However, it also has advantages such as allowing a high-flow injection and reducing the risk of catheter displacement [47]. At present, there is no consistent recommendation for the two catheters, and whether silicone catheters can be given priority for patients with a high risk of thrombosis needs further randomized controlled trials to determine.

There are two types of catheter ends: open and valve designs. Although prospective randomized controlled studies have shown that the short- and long-term complications caused by both are comparable [49]. It is generally believed that the design and operation of the catheter, such as its effects on blood flow dynamics and endothelial damage, play a significant role in thrombosis risk. Valved catheters might mitigate some of these risks through their design, which theoretically could reduce turbulence or endothelial injury, but further research is needed to confirm this. Meanwhile, the prolongation of PICC placement duration may elevate the risk of related venous thrombosis. This could be due to sustained irritation or damage to the venous endothelium from long-term catheter placement, partial venous obstruction leading to slowed blood flow, increased risk of infection, blood stagnation around the catheter area, and the possibility of the catheter surface becoming rough or microbial biofilm formation on the catheter surface, all of which may augment the risk of thrombus formation. As each patient’s situation may vary, the specific risk may differ individually. To mitigate PICC-related venous thrombosis risk, regular catheter maintenance and monitoring are crucial.

This study presents a comprehensive list of PICC-related venous thrombosis risk factors in cancer patients, encompassing specific patient characteristics, drug treatments, catheter features, and materials. This might be more thorough or detailed compared to existing literature, especially considering aspects like type (front-end opening catheter) and material (polyurethane). Additionally, the research particularly addressed the issue of multicollinearity in statistical analysis, offering new insights or further validating findings in existing literature. However, our research has some limitations. Firstly, further research is needed to verify the universality of our results, as the data is collected retrospectively. Secondly, although the results can help clinical doctors identify patients at high risk of thrombosis in a timely manner, they do not specifically recommend the treatment. We need multicenter data and treatment information to provide more definitive results and better guidance, and we will leave this for future work.

## Conclusions

We identified that the presence of age > 65 years old, male sex, white blood cell count > 9.5 × 10^9^ /L, APTT < 25 s, gastrointestinal tumor, infection, the use of cisplatin and vincristine, the use of polyurethane, open-ended catheters and keeping time of the catheter (days), were associated with PICC-related venous thrombosis.

## Declarations

### Availability of data and material

The datasets used and/or analyzed during the current study are available from the corresponding author on reasonable request.

## Abbreviations

APTT: Activated partial thromboplastin time
BMI: Body mass index
CDU: Color Doppler ultrasound
PICC: Peripherally inserted central catheter
VTE: Venous thromboembolism
